# A haplotype in the dipeptidyl peptidase 4 gene impacts glycemic-related traits of Brazilian older adults

**DOI:** 10.1590/1414-431X2022e12148

**Published:** 2022-10-03

**Authors:** E.S. Alves, A.C. Tonet-Furioso, V.P. Alves, C.F. Moraes, D.I.V. Pérez, I.M.D. Bastos, C. Córdova, O.T. Nóbrega

**Affiliations:** 1 Programa de Pós-Graduação em Ciências da Saúde, Faculdade de Medicina, Universidade de Brasília, Brasília, DF, Brasil; 2 Programa de Pós-Graduação em Gerontologia, Universidade Católica de Brasília, Taguatinga, DF, Brasil; 3 Kinesiology School, Physical Activity and Sports Science Master Program, Universidad Santo Tomás, Puerto Mont, Chile

**Keywords:** Dipeptidyl peptidase 4, Type 2 diabetes mellitus, Elderly, Glycemia

## Abstract

Dipeptidyl peptidase 4 (*DPP4*) regulates various physiological pathways and has a pivotal role in glucose homeostasis. The objective of this study was to verify the association of a haplotype constituted by two single nucleotide polymorphisms (*rs2268894* and *rs6741949*) in the *DPP4* gene with type 2 diabetes mellitus (T2DM) and fasting glycemia-related variables in a sample of Brazilian older adults, taking serum levels and enzymatic activity of DPP4 into account. Clinical, biochemical, and anthropometric characteristics as well as DPP4 serum levels and enzymatic activity were determined in 800 elderly (≥60 years old) individuals. Assessment of polymorphic sites was performed by real-time PCR whereas haplotypes were inferred from genotypic frequencies. Statistical analyses compared measures and proportions according to T2DM diagnosis and DPP4 haplotypic groups. The most common haplotype consisted of the T-rs2268894/G-rs6741949 string, which was 20% more frequent among non-diabetics. Considering non-diabetic patients alone, carriers of the T/G haplotype had significantly lower levels of blood glucose, insulin, HOMA-IR index, and DPP4 activity. Among diabetic patients, the T/G haplotype was associated with lower DPP4 levels whereas glycemic scores were not affected by allelic variants. Our results suggested that the genetic architecture of *DPP4* affects the glycemic profile and DPP4 serum levels and activity among elderly individuals according to the presence or absence of T2DM, with a possible implication of the T/G haplotype to the risk of T2DM onset.

## Introduction

Ageing entails numerous underlying physiological changes and a greater risk of chronic diseases (1). Type 2 diabetes mellitus (T2DM) is recognized as a global epidemic with considerable impact on personal and public health and is the ninth leading cause of death and seventh leading cause of disability worldwide in 2017 (2,3). T2DM is a chronic metabolic disorder characterized by a hyperglycemia status resulting primarily from the combination of progressive impairment of insulin secretion by pancreatic islet β-cells and insulin action on target tissues (4,5). This clinical condition is modulated by a wide range of modifiable (obesity, sedentary behavior, unhealthy diet) and non-modifiable (age and metabolic and genetic factors) contributors (5). Adults aged ≥65 years with T2DM account for around half of all individuals with diabetes (4).

Dipeptidyl peptidase 4 (DPP4, also known as CD26) is a ubiquitous serine protease seen both as a type II transmembrane glycoprotein in many cell types and as a soluble protein in plasma. DPP4 removes N-terminal X-proline and X-alanine dipeptides from several peptide substrates and regulates a variety of physiological pathways (6,7). DPP4 has a pivotal role in glucose homeostasis via rapid proteolytic inactivation of incretin hormones, namely glucagon-like peptide-1 (GLP-1) and glucose-dependent insulinotropic polypeptide (GIP), both responsible for ∼50% of postprandial insulin secretion (8). A 4-year follow-up study pointed out a greater DPP4 activity as an independent risk factor for prediabetes or type 2 diabetes among normoglycemic subjects at baseline (9). Although DPP4 activity and serum levels tend to be augmented in T2DM patients (7,10,11), other studies have found opposite results (12,13), leading to inconsistency in this regard.

Since the approval of DPP4 inhibitors for treatment of T2DM, the interest on alternate physiological effects of the enzyme and on context-sensitive association of DPP4 features with diabetes and other conditions has increased (8,10). Later, it became clear that DPP4 also has non-enzymatic functions as a binding and signaling protein, and that it stands out as an adipokine whose autocrine or paracrine action triggers insulin insensitivity (14,15). Thus, expression and activity levels of DPP4 have been found to be altered in conditions such as obesity (16), T2DM (17), and non-alcoholic fatty liver disease (NAFLD) (18), all of which indicate that analyses on DPP4 should take into account the context.

The human DPP4-encoding gene, located on chromosome 2q24.2 with 28 exons, contains almost 35,000 polymorphic sites, a few of which are associated with clinical conditions such as T2DM, myocardial infarction, dyslipidemia and coronavirus disease 2019 (COVID-19) (14, 19–21). Ahmed et al. (14) found single nucleotide polymorphisms (SNPs) of the *DPP4* gene not only associated with T2DM but also with DPP4 levels in Malaysian subjects, and a recent study suggests that the isolate alleles of two *DPP4* SNPs could be protective genetic markers for insulin resistance (IR) and hyperinsulinemia (22). In the same line, the *DPP4* rs6741949 polymorphism located in intron 2 has been shown to interact with body adiposity and negatively affect glucose-stimulated GLP-1 levels, insulin secretion, and glucose tolerance (23). On the other hand, there are contradictory findings regarding the association of the rs2268894 variants in the *DPP4* 3' untranslated region with the enzyme's activity, where SNP-dependent differences are occasionally observed (24,25). Overall, there is a body of evidence that alleles of *DPP4* are associated with either glycemic traits or expression/activity of its product, including this report on the association of rs6741949 SNP with parameters of insulin secretion (23) and another on the association of rs2268894 variation with DPP4 activity (25). Nonetheless, few studies have investigated the interplay between an assembly of variations on this gene and the T2DM phenotype in older adults (8).

Given the key role of DPP4 in glucose metabolism through the regulation of circulating levels of insulinotropic incretins and considering that T2DM is a multifactorial disease with a clear genetic component (26), the present study aimed to verify the association of a haplotype constituted by two common SNPs (rs2268894 and rs6741949) in non-coding regions of the *DPP4* gene with T2DM and fasting glycemia-related variables in a sample of Brazilian older adults, taking serum levels and enzymatic activity of DPP4 into account.

## Material and Methods

### Sample

The study sample included 800 non-institutionalized, unrelated patients aged 60 years or older recruited from geriatric outpatient clinics of the University of Brasília (UnB) and of the Catholic University of Brasília (UCB), Brazil. Inclusion criterion was to spontaneously seek primary or secondary care for circulatory events. Based on the study's nature, exclusion criteria encompassed T2DM therapy with DPP4 inhibitor or insulin, active and/or infectious inflammatory condition, neoplasia of any type (current or within the previous 2 years), and major kidney dysfunction (creatinine clearance <25 m · min^-1^· (1.73 m^2^)^-1^). No active search for patients with any specific condition was performed.

The study was approved by the Ethics Research Committee of the Faculty of Medicine (UnB, 24943719.8.0000.5558) and registered with the National System for Management of Genetic Heritage and Traditional Knowledge (SisGen, access code A407626). All participants signed a consent form before the beginning of data collection.

### Clinical evaluation

Clinical evaluation of patients was performed at both outpatient clinics following a standardized protocol. Systolic and diastolic blood pressure was measured according to current recommendations of the Brazilian Guidelines of Hypertension (27). Systemic arterial hypertension (SAH) was defined by systolic blood pressure (SBP) ≥130 mmHg, diastolic blood pressure (DBP) ≥85 mmHg, or antihypertensive use reported during clinical interview. Diagnosis of type 2 diabetes was determined by the presence of one of three criteria, as follows: glycated hemoglobin type-A1c (Hb1Ac) ≥6.5%, fasting plasma glucose ≥126 mg/dL, or use of antidiabetic drug(s) (28). Body mass index (BMI, in kg/m^2^) and waist circumference (WC, in cm) were evaluated with patients in light clothing and without shoes, with height measured with a wall-fixed stadiometer.

### Biochemical analyses

Biological samples were collected from peripheral blood of enrolled patients fasting for 8 to 12 h, with serum drawn and processed following routine laboratory analytical protocols after storage at -20°C for later batch analysis. Biochemical assays included determination of blood glucose, Hb1Ac, insulin, C-reactive protein (CRP), creatinine, and thyroid stimulating hormone (TSH). Tests were performed following routine clinical analyses with reagents from Boehringer Mannheim (Germany) and processed on AutoHumalyzer (Human GMBH, Germany) or nephelometer equipment (DadeBehring, Germany). Creatinine clearance (CC) was determined with the Cockcroft-Gault equation whereas the homeostatic model assessment of insulin resistance (HOMA-IR) index was calculated as fasting insulin × fasting glucose ÷ 22.5.

### DPP4 genotyping and haplotyping

Total genomic DNA was obtained using QIAamp DNA Mini Kit (Qiagen, Brazil), according to manufacturer's recommendations. Genotyping reactions were performed using real-time PCR (qPCR) TaqMan^®^ specific assays for the rs2268894 (T/C) and rs6741949 (C/G) SNPs of the *DPP4* gene with a universal master mix reagent, all from Thermo Fisher^®^ (USA). Settings for qPCR started with 50°C for 2 min and 95°C for 10 min followed by cycling conditions of 95°C for 15 s and 60°C for 1 min for 45 rounds, using the QuantStudio 3 Real-Time PCR System (Thermo Fisher^®^).

The strengths of linkage disequilibrium (LD) between the *DPP4* SNPs were determined using the Haploview software (Massachusetts Institute of Technology, USA), version 4.2 (29), with the haplotypes being inferred from the genotypic frequencies observed in the subjects based on the software-generated likelihood. Appearance in the haplotypes is ordered according to the genomic location (rs2268894-rs6741949) in the locus.

### DPP4 levels and enzymatic activity

Total circulating DPP4 was assessed by a specific enzyme-linked immunosorbent assay (ELISA) (R&D Systems, DuoSet, lot P185646), using a 1:1 dilution (1 part serum to 1 part saline) as sample and performed according to the manufacturer's instructions.

DPP4 serum enzymatic activity was determined as the rate of cleavage of 7-amino-4-methylcoumarin (AMC) from the fluorogenic substrate Glycyl-Prolyl-AMC (Gly-Pro-AMC; Sigma, USA) in a 96-well microplate in SpectraMax^®^ M5 ROM v3.0.22 Molecular Devices spectrofluorometer (USA) at 360 nm excitation and 460 nm emission for 15 min and in duplicate. The rate of liberated fluorescence was calculated per minute (U/min). All enzymatic reactions were performed with 10 µL of serum sample in activity buffer (25 mM HEPES, pH 7.5) in the presence of 20 µM of the substrate in a final volume of 100 µL (30). To confirm the specificity of DPP4 activity in these assays, a highly selective DPP4 inhibitor (Sitagliptin; Sigma) was incubated with some serum samples for 15 min before proceeding to hydrolysis of the substrate, in triplicate, with sitagliptin at a final concentration of 1 µM.

### Statistical analyses

Data are reported as means±SD or median and interquartile range according to data distribution assessed using the Kolmogorov-Smirnov test and normal probability plots. Student's *t*-test or one-way ANOVA was used for parametric data, whereas Mann-Whitney or Kruskal-Wallis was used for non-parametric data. Analyses of covariance (ANCOVA) was used to control statistics for confounding variables, with non-parametric data being log-transformed accordingly. The chi-squared test was used to compare proportions across groups. A linear regression model was used to test to which extent the T2DM phenotype was associated to *DPP4* haplotypes and to other non-glycemic traits (age, WC, SBP, TSH, CRP, and creatinine clearance), with any given haplotype rendered as a continuous trait by substituting its presence for 0, 1, or 2 as surrogate for amount.

The study power was calculated to detect a minimum net change of 0.4% in HbA1c levels. Considering that the overall standard deviation of the variable was in the order of 1.2%, the standardized difference was 0.3. Thus, 600 participants were required to detect such difference, with a power of 80%. Values of P≤0.05 on bilateral hypothesis tests were considered statistically significant. All statistical analyses were performed using SPSS for Windows version 18.0 (IBM, USA).

## Results

In this study, clinical, biochemical, anthropometric, and genomic aspects of a sample of 800 outpatients assisted in geriatric healthcare settings were determined. Type 2 diabetes mellitus was identified in 172 subjects (21.5%) of the study population, of which 97 (56.4%) were under antidiabetic pharmacotherapy, with metformin being the most prevalent drug. Of the 628 non-diabetic older adults, 201 had a glycemic profile compatible with pre-diabetes. Overall, inferential analyses revealed a sample of older adults with a rather homogeneous pattern of sociodemographic and clinical characteristics that did not differ significantly between carriers and non-carriers of T2DM, whether the latter were pre-diabetic or not, especially with respect to non-modifiable aspects such as age and sex and non-glycemic general traits such as blood pressure, kidney function, CRP, and TSH levels (Table 1). Even in the subset of T2DM patients, none of these traits (now including glycemic scores) were found to differ between user and non-users of antidiabetic drugs (data not shown), except for a slightly greater mean WC score among users (98.7 cm) compared to non-users (95.3 cm; P=0.034), with both scores (in treated and untreated T2DM) being significantly higher compared to that observed in the non-diabetic older adults (P=0.003 and P=0.028, in this order). Thus, greater body adiposity (expressed by higher BMI and WC scores) and increased CRP levels were obvious in the whole subset of patients with a severely disrupted glycemia homeostasis compared to the non-diabetic counterparts (Table 1), despite the intake of hypoglycemic drugs which, according to the reported clinical history, was ineffective to reach glycemic control. Overall, there was a high prevalence of metabolic disorders in the entire sample, indicated for instance by the slightly supra-physiological mean values of SBP and BMI regardless of diabetes diagnosis (31). Given that pre-diabetic older adults were comparable to the normoglycemic participants also in terms of HbA1c (P=0.152), insulin (P=0.084), and HOMA (P=0.095) indexes, except for an expected greater serum glycemia (P=0.001) markedly different from T2DM patients in glycemic profiles (P<0.001 for all these traits), pre-diabetic and normoglycemic older adults were merged into a single non-diabetic group for the purpose of providing power to the analyses.

**Table 1:** Comparison of clinical traits, fasting biochemistry, and haplotypic frequencies across non-diabetic and diabetic subjects.

	No T2DM (n=628)	T2DM (n=172)	P value
Age, years	73.8±9.2	74.8±9.1	0.206
WC, cm	92.4±11.8	97.4±9.8	<0.001
Gender, % male	33.0	32.6	0.920
BMI, kg/m^2^	26.6±4.6	28.1±4.6	<0.001
SBP, mmHg	140.6±23.3	142.9±22.8	0.279
DBP, mmHg	80.1±13.3	79.8±12.2	0.819
SAH, %	77.4	90.3	<0.001
Use of AHT drug, %	48.4	64.5	<0.001
CC, mL/min	61.5±24.0	65.7±26.3	0.106
TSH, mIU/L	2.1 [1.3, 3.4]	1.9 [1.5, 3.3]	0.603
CRP, mg/dL	1.2 [0.5, 2.6]	1.6 [0.9, 4.0]	0.027
HbA1c, %	5.6±0.6	6.9±1.8	<0.001
Glucose, mg/dL	95.6±13.5	138.5±54.1	<0.001
Insulin, mIU/L	6.6 [4.1, 10.4]	10.2 [6.6, 16.6]	<0.001
HOMA-IR, index	1.5 [0.9, 2.5]	3.5 [1.9, 5.3]	<0.001
Use of ADB drug, %	-	56.4	-
rs2268894 TT, %	31.0	22.7	0.023
rs6741949 GG, %	41.7	30.1	0.021
DPP4 level, ng/mL	2.4 [1.1, 4.4]	2.7 [1.4, 3.9]	0.072
DPP4 activity, 10^2^ U/min	2.4 [1.5, 3.8]	2.0 [1.2, 3.3]	0.015
DPP4 T/G haplotype, %	78.6	65.3	0.004

Data are reported as means±SD, within-group proportion (%), or median [interquartile range]. ADB: antidiabetic; AHT: antihypertensive; BMI: body mass index; CC: creatinine clearance; CRP: C-reactive protein; DBP: diastolic blood pressure; DPP4: dipeptidyl peptidase 4; HOMA-IR: Homeostatic Model Assessment of Insulin Resistance; HbA1c: glycated hemoglobin type-A1c; SAH: systemic arterial hypertension; SBP: systolic blood pressure; TSH: thyroid stimulating hormone; WC: waist circumference. Significance threshold set at P≤0.05. Student's *t*-test or one-way ANOVA were used for parametric data, whereas Mann-Whitney test or Kruskal-Wallis test were used for non-parametric data.

The genotypic frequencies obtained for each SNP were in Hardy-Weinberg equilibrium (χ^2^=0.71 for rs2268894 and =0.55 for rs6741949, P>0.05), and the association between genotypes and all clinical variables other than glycemia-related traits yielded non-significant results (P>0.05). Following a recessive model in a whole-group analysis, T2DM was less frequent among homozygotes for the major alleles T of rs2268894 and G of rs6741949 in the order of 26.8 and 27.8% respectively, when each genotype was considered separately. This justifies glycemic traits strongly unequally distributed between the two SNP since T2DM cases were less common in carriers of the TT-rs2268894 and GG-rs6741949 genotypes whereas all glycemic scores were increased in carriers of the other genotypes (data not shown). When assembled, these *DPP4* polymorphic sites were revealed to be in moderate LD in the sample (r^2^=0.48; D'=0.83) and showed different haplotypic structures by bioinformatics. The most frequent haplotype in the whole sample consisted of the T-rs2268894/G-rs6741949 string (49.9%), while other haplotypes identified were C/C (34.4%), C/G (12.2%), and T/C (3.5%). In complete agreement with the above findings from the SNPs taken individually, the T/G string was irregularly distributed according a T2DM diagnosis, being 20% more frequent in non-diabetic subjects compared to the diabetic counterparts (Table 1; P=0.004) and not associated with any of the non-glycemic variables evaluated. A linear regression analysis suggested that the T2DM phenotype exhibited a consistent relationship with the T/G haplotype, showing that the presence of any other haplotype, along with older age and greater WC, accounted for roughly half (∼50%) of the variance in the chance of a diabetes diagnosis (*β*=0.32, P=0.001).

**Figure 1:**
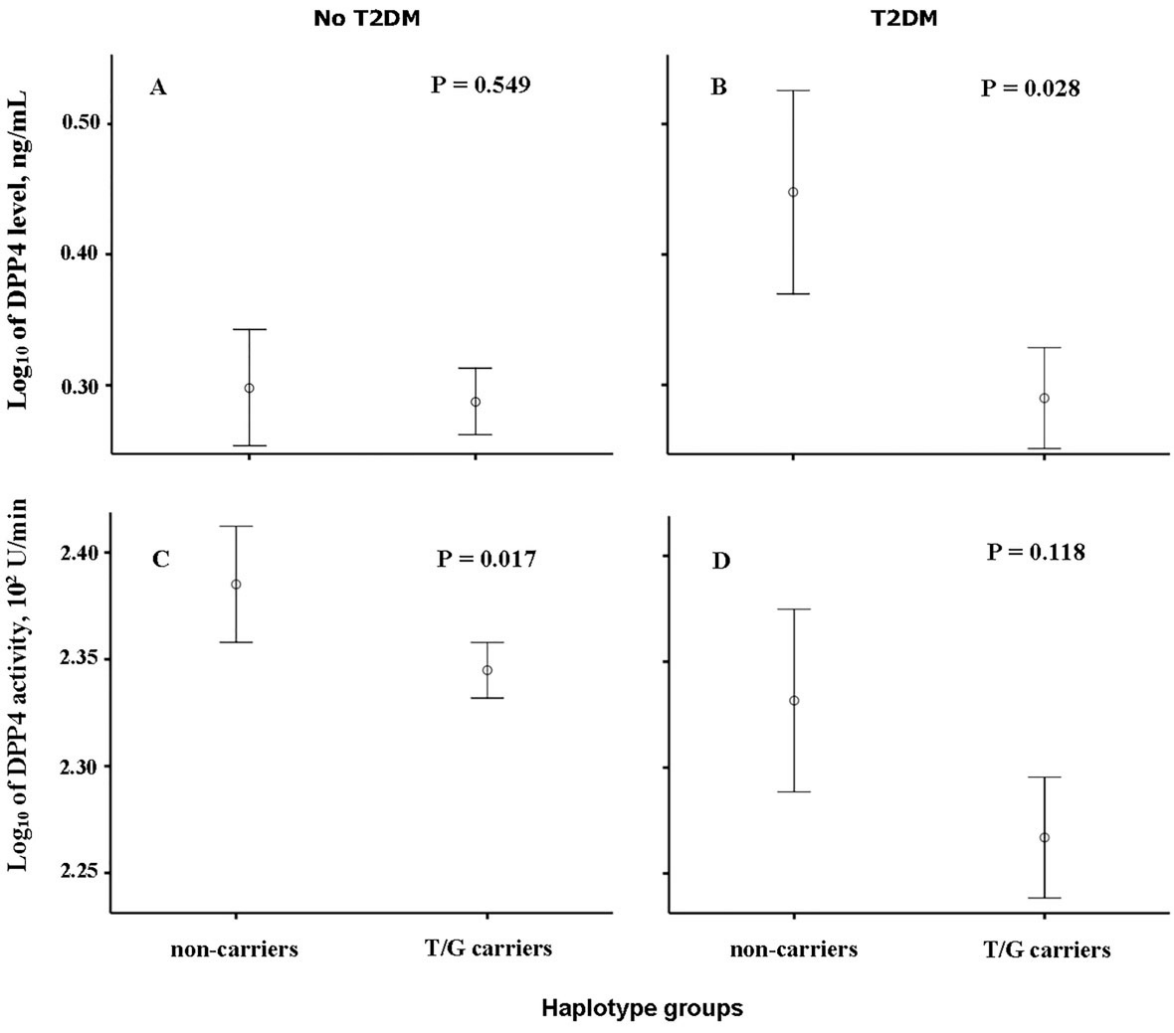
Comparison of log-transformed circulating levels (**A** and **B**) and log-transformed serum activity (**C** and **D**) of dipeptidyl peptidase 4 (DPP4) between carriers and non-carriers of the T/G haplotype of the gene, with individuals grouped according to a clinical diagnosis of type 2 diabetes mellitus (T2DM). Significance was verified by ANCOVA with adjustment for waist circumference and intake of antihypertensive agents (**A** and **C**) and for consumption of antidiabetic drugs (**B** and **D**). Data are reported as means and vertical bars represent intervals of one standard error.

Importantly, serum DPP4 activity was significantly lower in diabetic compared to non-diabetic subjects (P=0.015), while total circulating DPP4 levels did not differ among groups (P=0.072). Given that expression and activity levels of DPP4 vary greatly according to adiposity and insulin-resistance status, to explore a possible influence of the zygosity in T/G haplotype on glycemia-related variables in each of these scenarios patients were considered separately according to presence or absence of T2DM, as these groups had important differences in WC and BMI (Table 2). Among non-diabetic patients, carriers of the major DPP4 haplotype had significantly lower levels of blood glucose (P=0.003), insulin (P=0.034), and HOMA-IR index (P=0.007) along with a clear trend for lower glycated hemoglobin (P=0.053), while DPP4 activity was very reduced (P=0.046). This latter finding was corroborated by covariance analyses, in which the lower DPP4 activity of non-diabetic T/G carriers was confirmed even after adjustment for adiposity (WC) and number of antihypertensive agents taken (Figure 1). A remarkable null effect from haplotypes was observed in diabetic patients, as glycemic scores did not appear to be affected by variants (Table 2), although DPP4 levels were significantly affected by haplotypes (P=0.015). Also, the lower serum levels of DPP4 among diabetic T/G carriers was not influenced by the confounding variables mentioned above (now including the number of antidiabetic agents used) (Figure 1).

**Table 2:** Analysis of fasting glycemia-related variables according to the clinical condition of the subjects and haplotypes.

	Variable	Haplotype groups			P value
		other-other	T/G-other	T/G-T/G	
No T2DM		n=149	n=300	n=179	
	HbA1c, %	5.9±0.5	5.7±0.5	5.5±0.6	0.053
	Glucose, mg/dL	98.6±17.5	95.2±11.8	93.7±11.7	0.003
	Insulin, mIU/L	8.0 [4.8, 13.0]	6.5 [4.0, 10.3]	6.2 [3.9, 9.1]	0.034
	HOMA-IR, index	1.9 [1.1, 3.8]	1.5 [1.9, 2.4]	1.3 [0.9, 2.3]	0.007
	DPP4 level, ng/mL	2.5 [1.2, 4.7]	2.4 [1.1, 4.4]	2.3 [1.2, 4.0]	0.808
	DPP4 activity, 10^2^ U/min	260.0 [165.0, 402.5]	230 [155.0, 370]	215.0 [140.0, 360.0]	0.046
T2DM		n=61	n=81	n=30	
	HbA1c, %	7.0±1.7	6.8±1.6	6.7±2.3	0.734
	Glucose, mg/dL	145.1±54.9	136.5±58.1	131.7±42.2	0.492
	Insulin, mIU/L	9.7 [7.4, 19.8]	10.4 [6.2, 16.5]	7.2 [2.9, 14.9]	0.154
	HOMA-IR, index	3.5 [1.8, 5.7]	3.7 [1.9, 5.5]	2.2 [1.2, 4.0]	0.095
	DPP4 level, ng/mL	3.4 [1.8, 6.2]	2.6 [1.2, 3.1]	2.3 [1.5, 3.5]	0.015
	DPP4 activity, 10^2^ U/min	210.0 [138.8, 395.0]	175.0 [125.0, 285.0]	215.0 [132.5, 297.5]	0.285

Data are reported as means±SD or median [interquartile range]. DPP4: dipeptidyl peptidase 4; HOMA-IR: Homeostatic Model Assessment of Insulin Resistance; HbA1c: glycated hemoglobin type-A1c. Significance threshold set at P≤0.05. Student's *t*-test, Mann-Whitney test, or Kruskal-Wallis test were used.

## Discussion

T2DM is the result of a complex interaction between genetics and environment (5), and common genetic variations have been related to glucose metabolism disorders and T2DM pathogenesis (32,33). In this sense, variations of interest can also be found in the *DPP4* gene, a key player in glucose homeostasis (9), but the literature on their association with T2DM and glycemic traits remains elusive (8,14).

Since SNPs combined into haplotypes tend to be more informative than individual variations (34), we opted to investigate *DPP4* haplotypes constituted by the rs2268894 and rs6741949 alleles based on the assumption that SNPs placed at *locus* extremes and in moderate disequilibrium act as surrogates for other functional in-betweener genotypes for epidemiological purposes. Moreover, none of these SNPs are unevenly distributed across the main contributors to the Brazilian contingent (Europeans, Africans, and Amerindians) to reduce the risk of an ancestry-related bias in the findings.

In the total group analysis, the diabetic patients in our study presented reduced DPP4 activity compared to non-diabetics, regardless of body adiposity and CRP scores and despite the trend towards greater DPP4 levels in diabetics. Accordingly, some authors have found significantly lower DPP4 activity in individuals with T2DM, which is suggestive of an adaptive response of the diabetic body to a state of impaired responsiveness to insulin and in favor of an enhanced activity of enteroinsular hormones (12,24). These findings may also be due to the use of drugs such as metformin and glitazones by at least part of the diabetic patients, which may promote a decrease in DPP4 activity by still unknown mechanisms (6,11,35). More consistently, the T/G haplotype was found to be more frequent among non-diabetic subjects, possibly as a protective factor against T2DM onset.

Consistent with the above, our results showed that the influence of the DPP4 haplotypes on glycemic scores was context-sensitive. i.e., dependent on the presence or absence of T2DM (incidentally combined with adiposity), and that the T/G haplotype was associated with better glycemic scores among non-diabetic older adults. In this scenario, without major metabolic condition and without the influence of glycemia-acting agents, the finding that non-T/G carriers had high insulin levels despite having increased DPP4 activity is not surprising. The enzymatic activity of DPP4 correlates with its quaternary structure, and its dimeric structure is essential for optimal catalytic activity (8,36). It is possible that non-T/G isoforms homodimerize more efficiently, yielding a more active enzyme (11,37) that impairs incretin-dependent pathway(s) and promotes insulinotropic effect by (an) alternate but less effective route(s). Insulin levels may not be determined by incretins because macronutrients (especially amino acids and fatty acids) and other pancreatic hormones produced by α-cells (e.g., proglucagon-derived peptides) potentiate insulin secretion activated by a post-prandial increase in glucose concentration (38).

To the best of our knowledge, this is the first study to evaluate the DPP4 rs2268894 and rs6741949 polymorphisms in haplotype assembly, but other reports verified the repercussion of these SNPs individually. In line with our results, a previous study found that the C allele of rs6741949 SNP (within the non-T/G haplotypes) was associated with higher fasting glucose levels in subjects at increased risk for T2DM without difference in circulating DPP4 levels according to genotypes (DPP4 activity was not assessed) (23), and this could be because DPP4 is a ubiquitously expressed transmembrane protein and that our measurement was restricted to its proteolytically generated free circulating form.

Elevated circulating levels of DPP4 have already been reported In T2DM (8,17), possibly secondary to an increased shedding of DPP4 from adipocytes and peripheral blood mononuclear cells (35). According to our results, this appears to be a haplotype-dependent phenomenon since only non-T/G carriers displayed augmented DPP4 levels. A previous study demonstrated that metformin treatment consistently reduced levels of circulating DPP4 protein in humans with T2DM and cardiovascular disease, but it is uncertain whether this reduction is due to modulating DPP4 synthesis/shedding in specific tissues or due to DPP4 increased clearance (35). Therefore, it is also possible that metformin impairs DPP4 shedding only in the T/G carriers. Even so, DPP4 levels did not impact the enzyme's activity or glycemic traits, possibly due to the aforementioned pharmaceutical influence by antidiabetic drug use. To illustrate the heterogeneity in the literature, another report had shown that rs2268894 genotypes were not significantly associated with DPP4 activity or concentration in a sample of T2DM or hypertension carriers and healthy individuals overall (24).

The mechanism behind the association of the DPP4 haplotype with glycemic-related variables (as well as with enzyme levels and activity) assessed herein is not yet defined, since both SNPs are in noncoding regions. The functional impact of noncoding variants is hard to predict (39), and they are more likely to influence gene expression or mRNA stability, translation, localization, and access to regulators than the enzymatic function of the protein (23,40). Alterations of these regulatory mechanisms are known to modify molecular pathways and cellular processes, potentially inducing physiological changes and leading to disease processes (40). However, the hypothesis that the T/G haplotype is in linkage disequilibrium with functional polymorphisms in the *DPP4 locus* or neighboring sequences is more likely than a direct effect of the haplotype. Further studies are needed to identify these linked functional genotypes.

Limitations of the study include that unaccounted heterogeneities (e.g., genetic admixture) and a gene-environment interaction (eg., nutrition and lifestyle) may at least in part be responsible for inconsistences observed here and in the literature (26). Despite the effect of exercise and diet on glycemic profile and T2DM, the study did not evaluate levels of physical fitness and diet pattern among subjects. Also, markers of liver function were not evaluated, since liver diseases have already been associated with altered DPP4 levels and activity (13). In addition, the serum levels of active DPP4 substrates were not assessed and the possible effects of medications on glycemic traits and DPP4 levels and activity were not controlled for in our analyses.

In summary, our results corroborated that the genetic architecture of DPP4 affects the glycemic profile and DPP4 serum levels as well as activity among elderly individuals in a context-sensitive manner, according to the presence or absence of T2DM. In addition, T/G haplotype appeared protective against the development of T2DM not only by underscoring cases among its carriers but also by enhancing glycemic scores of non-diabetic older adults, possibly by preserving the incretin system and, consequently, insulin secretion via this route. Given that DPP4 is a multifunctional and ubiquitous protein that is important for numerous physiological processes, we suggest further studies with functional investigations to identify the physiological impact of genomic variations of *DPP4* in light of environmental factors with which they interact.
